# The analysis of main stressors among high-stress primary school teachers by job positions: A nationwide survey in Japan

**DOI:** 10.3389/fpubh.2022.990141

**Published:** 2022-12-22

**Authors:** Kenjiro Tsubono, Masaki Ogawa

**Affiliations:** ^1^Department of Psychosomatic Medicine, Tokai Central Hospital, Gifu, Japan; ^2^Department of Pharmacy, Tokai Central Hospital, Gifu, Japan

**Keywords:** teachers, stress responses, primary schools, stressors, working hours, co-worker relationship, interpersonal conflict

## Abstract

**Objectives:**

A school teacher's job is considered one of the most stressful occupations worldwide. To maintain the mental health of teachers, it is crucial to clarify the factors affecting work-related stress among teachers. The present study thus aimed to examine the main stressors among primary school teachers considering the difference in job positions by using data from a large-scale nationwide survey.

**Methods:**

We analyzed the data from a nationwide survey of public school teachers conducted between June and December 2021. The total number of participants was 138,651. The information of perceived main stressors, working hours per day, job workloads, job control, workplace support, and stress response scores were assessed by job position.

**Results:**

Among all teachers' job positions, the working hours of vice-principals were the longest, but their stress response scores were the second lowest. In contrast, the stress response scores among diet and nutrition teachers and health education teachers were the highest; their supervisors' and co-workers' support scores were the lowest among all teachers. Quantitative and qualitative workloads, job control, workplace support from supervisors and co-workers are significantly associated with teachers' stress responses in all job positions. Perceived main stressors among teachers were different depending on job positions. However, regardless of job positions, relationships with supervisors and co-workers were significantly associated with stress response scores among teachers. Dealing with difficult students and parents as well as workloads of clerical tasks were also associated with teachers' stress responses depending on job positions.

**Conclusions:**

Perceived main stressors among teachers were different depending on job positions. However, relationships with supervisors and co-workers were significantly associated with stress response levels among teachers regardless of job positions. This study highlighted the importance of interpersonal relationships at the workplace in terms of teachers' mental health. The results suggest that providing interpersonal skills training targeting co-workers' relationships and harassment prevention measures would be crucial to maintain teachers' mental health. The results also suggest that increasing school staff and providing sufficient organizational support for teachers will be required to prevent teachers' burnout.

## Introduction

Studies have shown that occupational stress is positively associated with employee's reduced performance, increased leave of absence, and turnover (1). Globally, teaching is considered one of the most stressful professions (2, 3). Previous studies have reported that school teachers have experienced a higher prevalence of psychological problems such as anxiety and depression (4, 5). Johnson et al. compared 26 different occupations and found that teachers exhibited the lowest level of psychological well-being and job satisfaction (6).

Teaching is regarded as a highly complicated, demanding task, requiring teachers to make prompt decisions in class (7). Teaching process involves a lot of emotional work with students (8). Prolonged negative experiences at work generate high level of emotional fatigue among teachers and negative attitudes toward their profession (9). A significant factor affecting teachers' attrition is burnout, which might result in them leaving the workplace (10). A school teacher is one of the occupations with the highest burnout rate (11). Teachers' occupational stress is linked to reduced job performance and increased burnout rate, which negatively affect students' academic achievements (7).

Research has revealed that teachers are exposed to various sources of stress. One of the major contributors to teachers' occupational stress is students' misbehavior. McCormick et al. reported student misbehavior as the biggest stressor related to teacher burnout (12). Studies investigating factors related to teachers' well-being have found that teachers consistently report poorer well-being when they encounter elevated levels of students' inattentiveness, classroom disturbances, or disciplinary problems (13, 14).

Working long hours is a long-standing issue among teachers across countries (15, 16). Working long hours is reported to be associated with psychological distress among school teachers (16, 17). Moreover, studies reported that high workload and time pressure are the main factors associated with occupational stress (18, 19). Apart from teaching duties, teachers are also burdened with a heavy load of administrative and clerical work, such as documentation and conducting programs. According to the results of the OECD Teaching and Learning International Survey conducted in 2018 (TALIS 2018), teachers experience higher levels of stress in their administrative work or school management duties than in the classroom (20).

Relationships with parents could be one of the primary sources of stress among teachers (21). The Office for Standards in Education, Children's Services and Skills reported that parents' unrealistic expectations and their excessive complaints considerably contributed to teachers' occupational stress (22). Stress from challenging parents caused low job satisfaction and even health problems among teachers (23).

It is noteworthy that interpersonal conflicts between co-workers are substantially associated with burnout in the workplace (24). The same is true of the school workplace: interpersonal workplace conflicts were positively associated with all dimensions of burnout among teachers (25). Conflict between principals and teachers could severely damage school climate and eventually affect students' academic achievements (26).

Generally, of the parameters influencing work-related stress, job demands and job control are two important factors, which are discussed in the Job Demands-Control (JDC) model developed by Karasek (27). “Job demand” in this model refers to workload and responsibilities placed on an individual employee. Job demands generally fall into two broad categories: quantitative workload (the amount and speed of work) and qualitative workload (cognitive, mental, and emotional efforts pertaining to the difficulty of tasks and an individual's capabilities) (28). Studies have reported that quantitative and qualitative workloads are associated with various mental problems among workers (29–31). Job control, the other stress factor in the JDC model, has also been reported to have a strong impact on an individual's perceived occupational stress (32, 33). According to the JDC model, the most stressful situation occurs for an employee when perceiving high job demands and low job control.

Increased attention to the buffering factors, such as workplace social support, emerged years after this model (34). The JDC model was extended to include workplace support (support from supervisors and co-workers) as a third predictive factor of employees' well-being; this extended model is known as the Job Demand-Control-Support (JDCS) model (35). Previous studies revealed that high levels of workplace support are associated with an individual's increased well-being and vice versa (36, 37). The study of Ibrahim et al. showed that job demands, job control and social support significantly affected teachers' psychological well-being (38). In addition, the effect of job demands on teachers' depression and anxiety was moderated by job control and social support (38). Therefore, it will be important to consider these factors in terms of teachers' work-related stress.

Various teachers' job positions and employment statuses exist in a school. For instance, teachers in administrative positions include principals or vice-principals and their duties are substantially different from those of class-room teachers. Accordingly, work-related stressors among teachers are expected to be different depending on job titles or positions. Therefore, it is necessary to address the differences in positions and related tasks to evaluate stressors among teachers.

Teachers in Japan have various duties to their students in addition to their essential educational work, such as clerical tasks, school management work, participation in training and research activities, Parent Teacher Association activities, and so forth. According to the results of TALIS 2018, the working hours of school teachers in Japan were the longest among the participating countries (20). In Japan, the percentage of school teachers taking leave due to mental illness has increased more than 5-fold from 0.11% in 1992 to 0.59% in 2019 (39).

In the Japanese education system, there are mainly six different teachers' positions. A principal and vice-principal are considered administrative positions. Tenured teachers are permanent contract teachers who have been accredited after passing the prefectural examination. Fixed-term teachers are those whose contracts need to be renewed annually. Health education teachers are teachers who offer education programs on illness prevention to students in addition to providing first aid to injured or sick students. Diet and nutrition teachers are responsible for the administration of the school lunch program, and providing students with knowledge about nutrition and a healthy diet.

In this context, to maintain the mental health of teachers, it is crucial to monitor their stress levels and clarify the factors affecting work-related stressors among teachers. To assess teachers' occupational stressors accurately and unbiasedly, a large-scale national-level survey covering a high percentage of the target population is necessary. Furthermore, considering the differences between job positions or roles is required for this purpose. However, insufficient consideration of the influence of job positions, in a nationwide survey with a high participation rate, currently exists.

In Japan, the Stress Check Program was implemented by the government in 2015, to prevent mental health problems in workers, requiring execution once a year in workplaces with 50 or more employees (40). In this program, employees' job stressors, and stress-related symptoms are assessed. More than 80% of the public primary school employees across the country have participated in this program in Japan.

The purpose of the present study is to assess primary school teachers' levels of occupational stress considering job positions and to clarify main stressors among high-stress teachers by using large-scale nationwide survey data. Finally, the study intends to offer a useful proposal for reducing teachers' occupational stress. For this purpose, we analyzed the data from the Stress Check Program conducted for public school employees across the country.

The research hypotheses are presented below:

Hypothesis 1–Regardless of job positions, job demands (quantitative and qualitative workloads), job control, workplace support from supervisors and co-workers are significantly associated with teachers' stress responses.

Hypothesis 2–Regardless of job positions, dealing with difficult students and parents, workloads of clerical tasks, school management duties, and relationship with supervisors and co-workers are significant sources of stress among high-stress teachers.

## 2. Materials and methods

### 2.1. Sample and data collection procedure

We used data from the Stress Check Program conducted between June and December 2021 by the Mutual Aid Association of Public School Teachers for public school employees across the country. The survey was conducted through a web-based questionnaire related to participants' characteristics, job positions, working hours per day, and work-related stressors. The total number of public primary school employees participating in the Stress Check program was 144,123 in 2021, which was 82.9% of all eligible employees. We obtained information regarding sex, age, job titles, employment status, working hours, stress response scores, and other stress factors. All participants were included in the analysis except for clerical workers. There were no participants whose data were missing. The total number of eligible participants were 131,029 (female = 80,423, 61.4%). As described above, there are mainly six different teachers' positions in the Japanese education system, which were included in the data utilized for this study. We analyzed and compared the data by each job-position category.

### 2.2. Measurements

#### 2.2.1. Working hours

We collected data about working hours per day, with seven response options as follows: (1) < 8 h (2) 8 to 9 h (3) 9 to 10 h (4) 10 to 11 h (5) 11 to 12 h (6) 12 to 13 h, and (7) 13 h or more. Due to the small number of participants working < 8 h, participants in the less-than-8 h and 8-to-9 h categories were combined into one group (< 9 h) for the data analysis.

#### 2.2.2. Stress response scores

In the Stress Check program, the Brief Job Stress Questionnaire (BJSQ) was used to evaluate teachers' stress levels. Several different language versions of the BJSQ are available for download (41). The BJSQ is an established questionnaire to identify high stress employees and is widely utilized in the field of occupational health in Japan (42, 43). The BJSQ was used to assess job stressors and stress responses in various occupations, such as teachers, nurses, physicians, and firefighters (44–47).

The BJSQ has adequate reliability and validity (48). It is a 57-item scale that assesses the following three aspects of work-related stressors: job demands and job control (17 items), psychological and physical stress responses (29 items), and buffering factors, such as co-worker support (11 items). In this study, the total score of the psychological and physical stress responses (29 items) was used for the analysis. Each item was rated on a four-point Likert scale (1 = almost never, 2 = sometimes, 3 = often, 4 = almost always). The scores of stress responses could range from 29 to 116, with higher scores indicating higher levels of stress. Of the 29 items, 18 were regarding psychological stress responses requiring responses on the following five dimensions: liveliness (3 items; e.g., “I have been lively”), irritability (3 items; e.g., “I have felt irritable”), fatigue (3 items; e.g., “I have felt extremely tired”), anxiety (3 items; e.g., “I have felt worried or insecure”), and depression (6 items; e.g., “I have been depressed”). The physical stress response was assessed by 11 questions on physical symptoms (e.g., “I have felt dizzy”). The total score of psychological and physical stress responses demonstrated high internal consistency (Cronbach's α = 0.90) (48). The stress response scores measured by the BJSQ were shown to predict a significant risk for the occurrence of depression (49). In this study, the total scores of psychological and physical stress responses were divided into quartiles, and participants in the top-quartile score were defined as “high stress,” according to the classification procedures in previous studies (16, 49).

#### 2.2.3. Quantitative and qualitative workloads, job control, and workplace support

To assess a participant's work-related stress, we also used the data of the following scales in the BJSQ: quantitative workload (e.g., “I have an extremely large amount of work to do”), qualitative workload (e.g., “I have to pay very careful attention”), job control (e.g., “I can choose how and in what order to do my work”), and supervisors' and co-workers' support (e.g., “How reliable are the following people when you are troubled?”) based on the theory of the JDCS model. These scales have demonstrated acceptable levels of validity and internal consistency (Cronbach's α = 0.82, 0.73, 0.76, and.79, respectively) (19, 50). Each item was rated on a four-point Likert like stress response scale, and the scores for each scale could range from 3 to 12 (each scale consists of 3 items). Higher scores indicate higher levels of workload for the quantitative and qualitative workload scales, and higher scores indicate higher levels of control over the work situation for the job-control scale. Regarding supervisors' and co-workers' support scales, higher scores indicate higher levels of support.

#### 2.2.4. Perceived main stressors of teachers

Participants were asked to choose their main stressors out of the following 14 items (up to two items could be selected): (1) responsibility for students' learning, (2) school management duties, (3) giving a demonstration lesson, (4) leading extra-curricular club activities (5) dealing with difficult students, (6) dealing with challenging parents, (7) workload of clerical tasks, (8) relationship with co-workers, (9) relationship with supervisors, (10) unfamiliar work environment due to a transfer, (11) long commuting time, (12) personal problems, (13) other problems, and (14) nothing in particular. The survey items on teachers' main stressors were selected by the Mutual Aid Association of Public School Teachers based on the opinions of psychiatrists and other mental health experts in affiliated hospitals. In this study, we investigated the main stressors among high-stress teachers for each job position.

### 2.3. Statistical analysis

Continuous variables were expressed as means (*M*) with standard deviation (*SD*) and medians (*Mdn*) with interquartile range (*IQR*); categorical variables were expressed as number of cases with percentages. The normality of distribution was assessed using the Kolmogorov-Smirnov test, and all continuous variables were found to deviate significantly from the normal distribution (*p* < 0.001). Differences in continuous variables were compared using the Mann-Whitney U test for two variables, and the Kruskal-Wallis test for more than three. To examine the association between job workloads, job control, supervisors' and co-workers' support, and stress responses, a multiple logistic regression analysis was performed for each job position by adjusting for the effects of sex and age. As described, the effects of job demands on teachers' occupational stress are expected to be moderated by job control and social support (38); therefore, we also assessed the effects of interactions between job workloads (quantitative and qualitative), job control and social support by including two-way interaction terms in the regression model. These variables were centered around their means before conducting the analysis.

For the statistical analysis of categorical variables, cross-tabulated frequencies and percentages were calculated. A chi-squared test was performed to examine the association between categorical valuables. As the sample size in this study was very large, we calculated the phi coefficient (Φ) for a 2 × 2 contingency table and Cramer's V for a larger than 2 × 2 contingency table as the effect size in addition to the *p*-value (51). Conventionally, Φ or Cramer's V value of < 0.1 was considered negligible, 0.1 a small effect, 0.3 a medium effect, and 0.5 a large effect (33). Accordingly, we interpreted the effect size's value of 0.1 as the minimum threshold of practical significance. All statistical analyses were performed using SPSS version 28 (IBM Corp., Armonk, NY, USA). The level of significance for each test was fixed at 0.05.

### 2.4. Ethical considerations

The study was conducted in accordance with the latest version of the Declaration of Helsinki, and was approved by the Institutional Review Board of Tokai Central Hospital (Reference No. 2022033101). This study used existing data for the study, and these data were already completely anonymized and untraceable. The ethics committee of the hospital ensured that all these procedures had been done properly, and made a judgement that informed consent was not required for the study.

## 3. Results

### 3.1. Participants' characteristics

Participants' descriptive statistics are shown in Table 1. For principals and vice-principals, the proportions of men were higher than those of women (76.5 and 69.7%, respectively), and most of the individuals in these administrative positions were aged 50 years or older (97.5% in principles and 78.0% in vice-principals). In contrast, most health education teachers and diet and nutrition teachers were women (99.5 and 97.1%, respectively).

**Table 1:** Participants' demographics.

	**Principal**		**Vice-principal**		**Tenured teacher**		**Fixed-term teacher**		**Health education teacher**		**Diet and nutrition teacher**		**Total**	
	***n***	**%**	***n***	**%**	***n***	**%**	***n***	**%**	***n***	**%**	***n***	**%**	***n***	**%**
Sex															
Male	5,354	76.5%	4,932	69.7%	36,546	38.1%	3,690	29.9%	33	0.5%	51	2.9%	50,606	38.6%
Female	1,644	23.5%	2,144	30.3%	59,445	61.9%	8,656	70.1%	6,822	99.5%	1,712	97.1%	80,423	61.4%
Age															
≤ 29	30	0.4%	8	0.1%	21,579	22.5%	3,497	28.3%	1,729	25.2%	345	19.6%	27,188	20.7%
30–39	37	0.5%	22	0.3%	23,481	24.5%	1,821	14.7%	1,430	20.9%	450	25.5%	27,241	20.8%
40–49	104	1.5%	1,521	21.5%	20,817	21.7%	2,098	17.0%	1,295	18.9%	478	27.1%	26,313	20.1%
50–59	5,670	81.0%	5,303	74.9%	22,848	23.8%	2,373	19.2%	1,959	28.6%	423	24.0%	38,576	29.4%
≥60	1,157	16.5%	222	3.1%	7266	7.6%	2,557	20.7%	442	6.4%	67	3.8%	11,711	8.9%

### 3.2. Comparisons of working hours by job position

**Figure 1:**
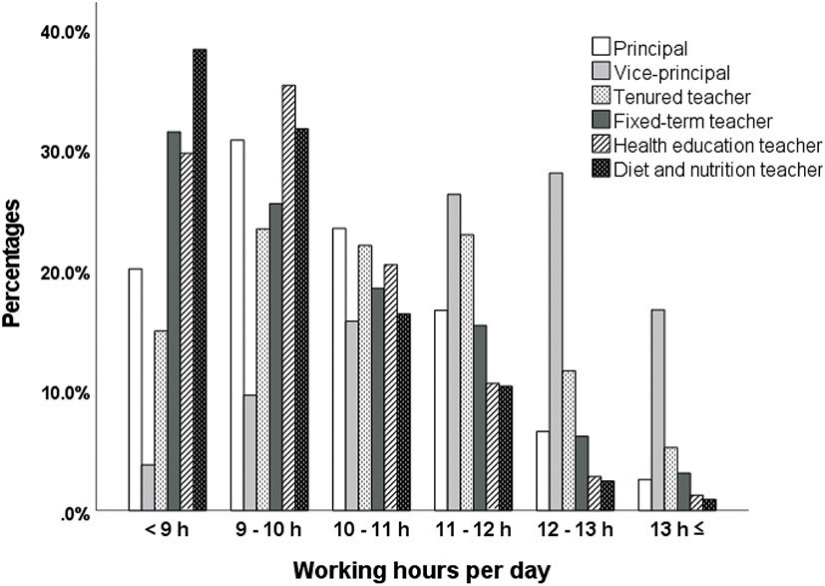
Comparisons of working hours per day by job position.

Figure 1 shows working hours per day for each job position. In the longest working-hour groups (11–12, 12–13, and ≥13 h), the percentages of vice-principals were the highest (26.2, 28.0, and 16.7%, respectively), followed by tenured teachers (22.9, 11.6, and 5.2%, respectively). On the other hand, in the shortest working-hour groups (< 9 h), the percentages of diet and nutrition teachers were the highest (38.3%), followed by fixed-term and health education teachers (31.4 and 29.7%, respectively). The results of the chi-squared test showed that the association between working hours and job positions was statistically significant (χ^2^ [25, *N* = 131,029] = 10963.838, *p* < 0.001, Cramer's V = 0.129).

### 3.3. Comparisons of job workload, job control, workplace support and stress response scores by job position

Table 2 shows the scores of workload, job control, workplace support, and stress response scales for each job position. The stress response scores of diet and nutrition teachers were the highest among all job positions (*Mdn* [*IQR*] = 57.0 [47.0-68.0]), followed by health education and tenured teachers (*Mdn* [*IQR*] = 55.0 [46.0-65.0] and 55.0 [45.0-65.0], respectively). In contrast, the stress response scores of principals were the lowest (*Mdn* [*IQR*] = 47.0 [40.0-57.0]), followed by vice-principals and fixed-term teachers (*Mdn* [*IQR*] = 51.0 [43.0-62.0] and 51.0 [42.0-62.0], respectively). Moreover, the Kruskal-Wallis test showed a significant difference in stress response scores between different job positions (χ^2^[5, *N* = 131,029] = 2063.128, *p* < 0.001).

**Table 2:** Comparison of the BJSQ job stress and stress response scores by job positions.

	**Principal (*****N*** = **6,998)**		**Vice–principal** **(*****N*** = **7,076)**		**Tenured teacher** **(*****N*** = **95,991)**	
	***M* (*SD*)**	***Mdn* (*IQR*)**	***M* (*SD*)**	***Mdn* (*IQR*)**	***M* (*SD*)**	***Mdn* (*IQR*)**
Quantitative workload^a^	8.4 (1.86)	8.0 (7.0–9.0)	9.8 (1.76)	10.0 (9.0–11.0)	9.7 (1.89)	10.0 (9.0–11.0)
Qualitative workload^a^	9.4 (1.73)	9.0 (8.0–11.0)	9.4 (1.61)	9.0 (8.0–11.0)	9.4 (1.72)	9.0 (8.0–11.0)
Job control^b^	9.6 (1.58)	9.0 (9.0–11.0)	8.0 (1.75)	8.0 (7.0–9.0)	8.0 (1.79)	8.0 (7.0–9.0)
Supervisors' support^c^	—	9.0 (7.0–10.0)	9.5 (2.17)	10.0 (8.0–12.0)	8.5 (2.24)	9.0 (7.0–10.0)
Co–workers' support^c^	9.0 (1.87)	9.0 (8.0–10.0)	9.0 (1.99)	9.0 (8.0–10.0)	9.1 (2.04)	9.0 (8.0–11.0)
Stress response scores	49.5 (12.89)	47.0 (40.0–57.0)	53.4 (14.13)	51.0 (43.0–62.0)	56.3 (14.94)	55.0 (45.0–65.0)
	**Fixed–term teacher** **(*****N*** = **12,346)**		**Health education teacher** **(*****N*** = **6,855)**		**Diet and nutrition teacher** **(*****N*** = **1,763)**	
	***M*** **(*****SD*****)**	***Mdn*** **(*****IQR*****)**	***M*** **(*****SD*****)**	***Mdn*** **(*****IQR*****)**	***M*** **(*****SD*****)**	***Mdn*** **(*****IQR*****)**
Quantitative workload^a^	8.7 (2.12)	9.0 (7.0–10.0)	8.7 (1.91)	9.0 (8.0–10.0)	9.3 (1.84)	9.0 (9.0–11.0)
Qualitative workload^a^	9.0 (1.82)	9.0 (8.0–10.0)	8.8 (1.68)	9.0 (8.0–10.0)	9.1 (1.58)	9.0 (8.0–10.0)
Job control^b^	8.0 (1.85)	8.0 (7.0–9.0)	8.8 (1.65)	9.0 (8.0–10.0)	8.8 (1.71)	9.0 (8.0–9.0)
Supervisors' support^c^	8.5 (2.24)	9.0 (7.0–10.0)	8.3 (2.15)	8.0 (7.0–10.0)	7.9 (2.15)	8.0 (6.0–9.0)
Co–workers' support^c^	9.1 (2.11)	9.0 (8.0–11.0)	8.6 (2.01)	9.0 (7.0–10.0)	8.3 (2.06)	8.0 (7.0–9.0)
Stress response scores	53.5 (14.89)	51.0 (42.0–62.0)	56.1 (14.03)	55.0 (46.0–65.0)	58.0 (14.81)	57.0 (47.0–68.0)

BJSQ, Brief Job Stress Questionaries; SD, standard deviation; IQR, interquartile range.

^a^Scores range from 3.0 and 12.0 with higher scores indicating higher job stress.

^b^Scores range from 3.0 and 12.0 with higher scores indicating higher job control.

^c^Scores range from 3.0 and 12.0 with higher scores indicating higher social support.

The scores of quantitative workload were the highest among vice-principals and tenured teachers (*Mdn* [*IQR*] = 10.0 [9.0-11.0] for both) and the lowest among principals (*Mdn* [*IQR*] = 8.0 [7.0-9.0]). The scores of job control were the highest among principals (*Mdn* [*IQR*] = 10.0 [9.0-11.0]), and lowest in vice-principals, tenured teachers, and fixed-term teachers (*Mdn* [*IQR*] = 8.0 [7.0-9.0] for all of them). The scores of supervisors' support were the highest among vice-principals (*Mdn* [*IQR*] = 10.0 [8.0-12.0]) and the lowest in health education and diet and nutrition teachers (*Mdn* [*IQR*] = 8.0 [7.0-10.0] and 8.0 [6.0-9.0], respectively). The scores of co-workers' support were the lowest among diet and nutrition teachers (*Mdn* [*IQR*] = 8.0 [7.0-9.0]).

### 3.4. Stress response scores in each working-hour category by job position

**Figure 2:**
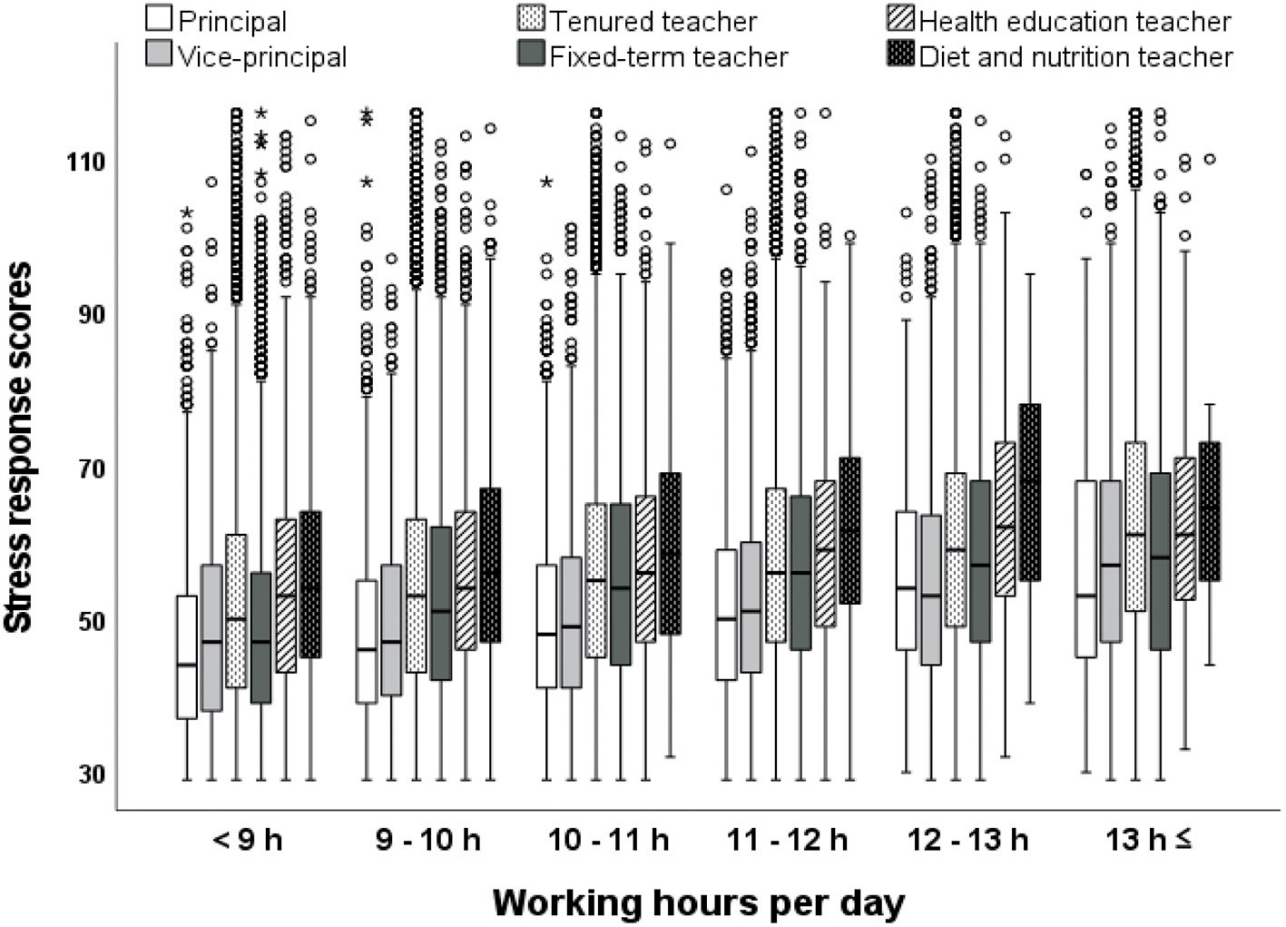
Comparisons of stress response scores in each working-hour group by job position.

Figure 2 shows the stress response scores in each working-hour category by job position. The results revealed that the stress response scores increased as the working hours per day became longer in all job positions. The Kruskal-Wallis test showed a significant difference in stress response scores between different working-hour categories in all job positions (*p* < 0.001). In the same working-hour category, the stress response scores of diet and nutrition teachers were the highest, followed by health education and tenured teachers. In contrast, the stress response scores of principals and vice-principals were the lowest in all working-hour categories.

### 3.5. Relationship between job workload, job control, workplace support, and stress responses by job position

Table 3 shows the results of the multiple logistic regression analysis examining the association between job workload, job control, workplace support and stress responses, adjusting for the effects of sex and age, without considering interaction effects between variables. All of these scales were significantly associated with stress responses in all job positions (*p* < 0.001). Among tenured and fixed-term teachers, females exhibited significantly higher stress responses than males (OR [95% CI] = 1.26 [1.22–1.30], *p* < 0.001, and 1.12 [0.99–1.25], *p* = 0.007 respectively). In all job positions except for vice-principals, participants in higher age groups showed significantly lower stress responses.

**Table 3:** Relationship between the BJSQ job stress and stress response scores among primary school teachers (logistic regression analysis adjusted for sex and age without interaction terms).

	**Principal**			**Vice–principal**			**Tenured teacher**		
	**OR**	**95% CI**	***p***	**OR**	**95% CI**	***p***	**OR**	**95% CI**	***p***
Sex (Ref. Male)	1.11	0.93–1.33	0.245	1.13	0.98–1.31	0.097	1.26	1.22–1.30	< 0.001
Age (Ref. 30–39 years)									
≤ 29 years	0.35	0.09–1.35	0.127	0.00	0.00	0.999	1.01	0.97–1.06	0.598
40–49 years	0.22	0.08–0.65	0.006	0.59	0.19–1.78	0.347	0.88	0.84–0.92	< 0.001
50–59 years	0.30	0.13–0.69	0.005	0.52	0.17–1.57	0.245	0.82	0.78–0.86	< 0.001
≥ 60 years	0.24	0.10–0.58	0.001	0.61	0.19–1.96	0.407	0.55	0.51–0.60	< 0.001
Quantitative workload^a^	1.28	1.20–1.35	< 0.001	1.29	1.22–1.37	< 0.001	1.27	1.26–1.29	< 0.001
Qualitative workload^a^	1.36	1.27–1.45	< 0.001	1.29	1.22–1.37	< 0.001	1.30	1.28–1.32	< 0.001
Job control^b^	0.80	0.76–0.84	< 0.001	0.70	0.67–0.74	< 0.001	0.77	0.76–0.77	< 0.001
Supervisors' support^c^		—		0.87	0.84–0.91	< 0.001	0.91	0.90–0.82	< 0.001
Co–workers' support^c^	0.73	0.69–0.77	< 0.001	0.80	0.77–0.84	< 0.001	0.85	0.84–0.86	< 0.001
Nagelkerke *R*^2^		0.309			0.339			0.288	
	**Fixed–term teacher**			**Health education teacher**			**Diet and nutrition teacher**		
	**OR**	**95% CI**	***p***	**OR**	**95% CI**	***p***	**OR**	**95% CI**	***p***
Sex (Ref. Male)	1.12	0.99–1.25	0.007	1.50	0.60–3.78	0.385	0.54	0.28–1.07	0.075
Age (Ref. 30–39 years)									
≤ 29 years	1.04	0.90–1.21	0.588	1.17	0.98–1.40	0.086	0.96	0.68–1.35	0.820
40–49 years	0.71	0.60–0.83	< 0.001	0.80	0.66–0.98	0.027	0.67	0.49–0.92	0.013
50–59 years	0.59	0.50–0.70	< 0.001	0.91	0.76–1.08	0.268	0.77	0.56–1.06	0.112
≥ 60 years	0.43	0.36–0.51	< 0.001	0.60	0.44–0.81	< 0.001	0.49	0.25–0.95	0.035
Quantitative workload^a^	1.27	1.23–1.31	< 0.001	1.25	1.20–1.30	< 0.001	1.31	1.20–1.42	< 0.001
Qualitative workload^a^	1.30	1.25–1.35	< 0.001	1.24	1.18–1.30	< 0.001	1.30	1.18–1.44	< 0.001
Job control^b^	0.78	0.76–0.80	< 0.001	0.79	0.76–0.83	< 0.001	0.79	0.73–0.85	< 0.001
Supervisors' support^c^	0.89	0.87–0.92	< 0.001	0.86	0.82–0.89	< 0.001	0.92	0.86–0.99	0.020
Co–workers' support^c^	0.85	0.83–0.88	< 0.001	0.83	0.80–0.87	< 0.001	0.82	0.76–0.88	< 0.001
Nagelkerke *R*^2^		0.298			0.272			0.281	

A dependent variable is “high–stress” teachers vs. others. “High stress” represents participants whose stress response scores are in the top quartile.

BJSQ, Brief Job Stress Questionaries; OR, odds ratio; CI, confidence intervals.

^a^Scores range from 3.0 and 12.0 with higher scores indicating higher job stress.

^b^Scores range from 3.0 and 12.0 with higher scores indicating higher job control.

^c^Scores range from 3.0 and 12.0 with higher scores indicating higher social support.

Table 4 shows the results of the same logistic regression model including two-way interaction terms between job control, workplace support and job workload. For tenured and fixed-term teachers, interaction effects between quantitative workload and co-workers' support were statistically significant (*p* < 0.001). It represents that the effects of quantitative workload on teachers' stress responses were moderated by co-workers' support. To assess the interaction effects between these two variables, we conducted a subgroup analysis in which tenured and fixed-term teachers were divided into four subgroups depending on the scores of co-workers' support (from the first quartile to the top quartile). The multiple logistic regression analysis adjusting for the effects of age and sex was performed for each subgroup. Table 5 shows the odds ratio expressing the association between quantitative workload and high stress responses at the different levels of co-workers' support. The results revealed that the odds ratio representing this association increased as the levels of co-workers' support became higher. No other interaction effects were statistically significant in all job positions.

**Table 4:** Relationship between the BJSQ job stress and stress response scores among primary school teachers (logistic regression analysis adjusted for sex and age with interaction terms).

	**Principal**			**Vice–principal**			**Tenured teacher**		
	**OR**	**95% CI**	***p***	**OR**	**95% CI**	***p***	**OR**	**95% CI**	***p***
Sex (Ref. Male)	1.15	0.96–1.37	0.124	1.13	0.98–1.31	0.092	1.26	1.22–1.31	< 0.001
Age (Ref. 30–39 years)									
≤ 29 years	0.31	0.08–1.22	0.093	0.00	0.00	0.999	1.01	0.97–1.06	0.580
40–49 years	0.22	0.08–0.64	0.005	0.57	0.19–1.75	0.328	0.88	0.84–0.92	< 0.001
50–59 years	0.30	0.13–0.69	0.005	0.51	0.17–1.54	0.230	0.82	0.78–0.86	< 0.001
≥ 60 years	0.25	0.11–0.60	0.002	0.60	0.18–1.94	0.389	0.54	0.50–0.59	< 0.001
Quantitative workload^a^	1.27	1.19–1.35	< 0.001	1.28	1.18–1.38	< 0.001	1.27	1.25–1.29	< 0.001
Qualitative workload^a^	1.36	1.27–1.45	< 0.001	1.34	1.22–1.46	< 0.001	1.29	1.27–1.31	< 0.001
Job control^b^	0.78	0.73–0.84	< 0.001	0.69	0.65–0.73	< 0.001	0.77	0.76–0.78	< 0.001
Supervisors' support^c^		—		0.84	0.80–0.89	< 0.001	0.91	0.89–0.92	< 0.001
Co–workers' support^c^	0.64	0.60–0.69	< 0.001	0.78	0.74–0.83	< 0.001	0.84	0.34–0.85	< 0.001
Quantitative workload × job control	0.97	0.94–1.01	0.146	1.00	0.96–1.03	0.897	1.00	0.99–1.01	0.686
Qualitative workload × job control	1.01	0.96–1.05	0.739	1.03	0.99–1.06	0.170	0.99	0.99–1.00	0.146
Quantitative workload × supervisors' support		—		1.02	0.99–1.05	0.187	1.00	1.00–1.01	0.412
Quantitative workload × co–workers' support	0.99	0.96–1.02	0.441	1.03	0.99–1.06	0.095	1.02	1.01–1.02	< 0.001
Qualitative workload × supervisors' support		—		1.01	0.98–1.04	0.532	0.99	0.99–1.01	0.591
Qualitative workloads × co–workers' support	1.00	0.96–1.32	0.833	0.99	0.96–1.03	0.637	1.00	0.99–1.01	0.929
Nagelkerke *R*^2^		0.299			0.343			0.288	
	**Fixed–term teacher**			**Health education teacher**			**Diet and nutrition teacher**		
	**OR**	**95% CI**	***p***	**OR**	**95% CI**	***p***	**OR**	**95% CI**	***p***
Sex (Ref. Male)	1.12	0.99–1.26	0.054	1.53	0.61–3.88	0.366	0.53	0.27–1.05	0.530
Age (Ref. 30–39 years)									
≤ 29 years	1.04	0.90–1.20	0.610	1.16	0.97–1.39	0.100	0.97	0.69–1.37	0.870
40–49 years	0.71	0.60–0.83	< 0.001	0.80	0.66–0.97	0.026	0.68	0.49–0.93	0.015
50–59 years	0.59	0.50–0.70	< 0.001	0.91	0.76–1.08	0.263	0.77	0.56–1.08	0.127
≥ 60 years	0.43	0.36–0.51	< 0.001	0.60	0.44–0.81	< 0.001	0.49	0.25–0.95	0.034
Quantitative workload^a^	1.26	1.21–1.31	< 0.001	1.24	1.19–1.30	< 0.001	1.33	1.21–1.45	< 0.001
Qualitative workload^a^	1.31	1.25–1.38	< 0.001	1.24	1.18–1.30	< 0.001	1.31	1.18–1.45	< 0.001
Job control^b^	0.78	0.75–0.80	< 0.001	0.79	0.76–0.83	< 0.001	0.76	0.70–0.83	< 0.001
Supervisors' support^c^	0.90	0.87–0.93	< 0.001	0.86	0.82–0.89	< 0.001	0.32	0.86–1.01	0.066
Co–workers' support^c^	0.85	0.82–0.88	< 0.001	0.83	0.79–0.86	< 0.001	0.81	0.75–0.88	< 0.001
Quantitative workload × job control	1.00	0.98–1.01	0.676	0.99	0.96–1.02	0.445	0.99	0.94–1.05	0.790
Qualitative workload × job control	1.01	0.99–1.03	0.342	0.99	0.96–1.02	0.631	1.05	0.99–1.12	0.107
Quantitative workload × supervisors' support	0.99	0.98–1.01	0.574	0.99	0.96–1.01	0.261	1.00	0.95–1.05	0.861
Quantitative workload × co–workers' support	1.03	1.01–1.05	0.003	1.01	0.98–1.04	0.519	1.02	0.97–1.08	0.358
Qualitative workload × supervisors' support	0.99	0.97–1.01	0.450	1.00	0.97–1.03	0.897	0.99	0.93–1.05	0.729
Qualitative workloads × co–workers' support	0.99	0.97–1.01	0.428	1.02	0.99–1.05	0.293	1.00	0.94–1.07	0.956
Nagelkerke *R*^2^		0.273			0.284			0.309	

A dependent variable is “high–stress” teachers vs. others. “High stress” represents participants whose stress response scores are in the top quartile.

BJSQ, Brief Job Stress Questionaries; OR, odds ratio; CI, confidence intervals.

^a^Scores range between 3.0 and 12.0 with higher scores indicating higher workloads.

^b^Scores range between 3.0 and 12.0 with higher scores indicating higher job control.

^c^Scores range between 3.0 and 12.0 with higher scores indicating higher social support.

**Table 5:** Odds ratios expressing the association between quantitative workloads and high stress responses at the different levels of co–workers' support (adjusting for the effects of sex and age).

	**Tenured teacher**			**Fixed–term teacher**		
**Level of co–workers' support^a^**	**OR**	**95% CI**	***p***	**OR**	**95% CI**	***p***
First quartile (scores^b^ < 8.0)	1.21	1.19–1.24	< 0.001	1.19	1.13–1.26	< 0.001
Second quartile (8.0 ≤ scores < 9.0)	1.26	1.24–1.27	< 0.001	1.25	1.21–1.29	< 0.001
Third quartile (9.0 ≤ scores < 11.0)	1.26	1.24–1.27	< 0.001	1.25	1.21–1.29	< 0.001
Top quartile (11.0 ≤ scores)	1.29	1.25–1.33	< 0.001	1.33	1.23–1.43	< 0.001

^a^Participants were divided into four different subgroups depending on co–workers' support scores.

^b^Scores means co–workers' support scores ranging between 3.0 and 12.0. Higher scores indicate higher levels of co–workers' support.

### 3.6. Perceived main sources of stress among high-stress teachers

Table 6 shows the association between perceived main sources of stress and stress response scores among teachers. A very small percentage of participants (< 1.0%) selected “extra-curricular club activities” as a main stressor; therefore, it was excluded from the statistical analysis. The percentages of high-stress participants were the highest among diet and nutrition teachers (30.4%), followed by health education and tenured teachers (25.5 and 26.8%, respectively). In contrast, the percentages of high-stress participants were the lowest among principals (12.8%), followed by vice-principals and fixed-term teachers (19.9 and 21.6%, respectively).

**Table 6:** Association between main stressors and stress response scores among primary school teachers.

		**Principal**		**Vice–principal**		**Tenured teacher**	
		**High stress (*N* = 896)**	**Others (*N* = 6,102)**	**High stress (*N* = 1,408)**	**Others (*N* = 5,668)**	**High stress (*N* = 25,763)**	**Others (*N* = 70,228)**
Relationship with supervisors	Count (%)	—	—	232 (16.2%)	259 (5.0%)	2,755 (10.7%)	2,895 (4.1%)
	Φ	—		0.187**		0.124**	
Relationship with co–workers	Count (%)	243 (27.1%)	710 (11.6%)	290 (20.6%)	613 (10.8%)	3,767 (14.6%)	5,129 (7.3%)
	Φ	0.151**		0.117**		0.112**	
Dealing with challenging parents	Count (%)	376 (42.0%)	1,663 (27.3%)	387 (27.5%)	1,223 (21.6%)	4,740 (18.4%)	8,749 (12.5%)
	Φ	0.108**		0.056**		0.076**	
Dealing with difficult students	Count (%)	254 (28.3%)	1,279 (21.0%)	248 (17.6%)	884 (15.6%)	8,716 (33.8%)	15,821 (22.5%)
	Φ	0.060**		0.022		0.115**	
Workload of clerical tasks	Count (%)	128 (14.3%)	429 (7.0%)	702 (49.9%)	1,901 (33.5%)	6,252 (24.3%)	13,219 (18.8%)
	Φ	0.090**		0.135**		0.060**	
Unfamiliar work environment	Count (%)	84 (9.4%)	271 (4.4%)	179 (12.7%)	368 (6.5%)	1,836 (7.1%)	3,102 (4.4%)
	Φ	0.075**		0.093**		0.054**	
Responsibility for students' learning	Count (%)	7 (0.8%)	51 (0.8%)	19 (1.3%)	46 (0.8%)	3,633 (14.1%)	7,310 (10.4%)
	Φ	−0.002		0.023		0.051**	
School management duties	Count (%)	45 (5.0%)	182 (3.0%)	107 (7.6%)	283 (5.0%)	4,839 (18.8%)	9,562 (13.6%)
	Φ	0.038*		0.046**		0.064**	
Demonstration lessons	Count (%)	6 (0.7%)	25 (0.4%)	5 (0.4%)	14 (0.2%)	2,555 (9.9%)	5,528 (7.9%)
	Φ	0.013		0.008		0.033**	
Long commuting time	Count (%)	45 (5.0%)	262 (4.3%)	62 (4.4%)	286 (5.0%)	974 (3.8%)	2,599 (3.7%)
	Φ	0.012		−0.012		0.002	
Personal problems	Count (%)	133 (14.8%)	682 (11.2%)	168 (11.9%)	494 (8.7%)	3,262 (12.7%)	6,729 (9.6%)
	Φ	0.038*		0.044**		0.045**	
Other problems	Count (%)	126 (14.1%)	513 (8.4%)	95 (6.7%)	227 (4.0%)	1,263 (4.9%)	2,367 (3.4%)
	Φ	0.066**		0.053**		0.036**	
Nothing	Count (%)	46 (5.1%)	2,014 (33.0%)	36 (2.6%)	1,543 (27.2%)	1,066 (4.1%)	18,894 (26.9%)
	Φ	−0.204**		−0.237**		−0.249**	
		**Fixed–term teacher**		**Health education teacher**		**Diet and nutrition teacher**	
		**High stress (*****N*** = **2,662)**	**Others (*****N*** = **9,684)**	**High stress (*****N*** = **1,751)**	**Others (*****N*** = **5,104)**	**High stress (*****N*** = **536)**	**Others (*****N*** = **1,227)**
Relationship with supervisors	Count (%)	282 (10.6%)	316 (3.3%)	330 (18.8%)	310 (6.1%)	70 (13.1%)	70 (5.7%)
	Φ	0.140**		0.191**		0.125**	
Relationship with co–workers	Count (%)	433 (16.3%)	596 (6.2%)	365 (20.8%)	493 (9.7%)	137 (25.6%)	159 (13.0%)
	Φ	0.150**		0.147**		0.155**	
Dealing with challenging parents	Count (%)	447 (16.8%)	814 (8.4%)	140 (8.0%)	295 (5.8%)	17 (3.2%)	20 (1.6%)
	Φ	0.114**		0.040*		0.049*	
Dealing with difficult students	Count (%)	903 (33.9%)	1,864 (19.2%)	405 (23.1%)	882 (17.3%)	7 (1.3%)	18 (1.5%)
	Φ	0.145**		0.065**		0.006	
Workload of clerical tasks	Count (%)	411 (15.4%)	896 (9.3%)	410 (23.4%)	830 (16.3%)	243 (45.3%)	358 (29.2%)
	Φ	0.083**		0.081**		0.157**	
Unfamiliar work environment	Count (%)	239 (9.0%)	406 (4.2%)	218 (12.5%)	416 (8.2%)	77 (14.4%)	95 (7.7%)
	Φ	0.088**		0.065**		0.103**	
Responsibility for students' learning	Count (%)	483 (18.1%)	1,167 (12.1%)	6 (0.3%)	11 (0.2%)	5 (0.9%)	14 (1.1%)
	Φ	0.074**		0.011		−0.009	
School management duties	Count (%)	245 (9.2%)	566 (5.8%)	256 (14.6%)	468 (9.2%)	40 (7.5%)	58 (4.7%)
	Φ	0.056**		0.077**		0.055*	
Demonstration lessons	Count (%)	204 (7.7%)	533 (5.5%)	29 (1.7%)	78 (1.5%)	12 (2.2%)	28 (2.3%)
	Φ	0.037**		0.005		−0.001	
Long commuting time	Count (%)	98 (3.7%)	303 (3.1%)	117 (6.7%)	320 (6.3%)	48 (9.0%)	96 (7.8%)
	Φ	0.013		0.007		0.019	
Personal problems	Count (%)	368 (13.8%)	1,036 (10.7%)	425 (24.3%)	900 (17.6%)	111 (20.7%)	163 (13.3%)
	Φ	0.040**		0.073**		0.094**	
Other problems	Count (%)	136 (5.1%)	323 (3.3%)	181 (10.3%)	353 (6.9%)	63 (11.8%)	92 (7.5%)
	Φ	0.039**		0.056**		0.069*	
Nothing	Count (%)	246 (9.2%)	3,854 (39.8%)	107 (6.1%)	1,564 (30.6%)	39 (7.3%)	409 (33.3%)
	Φ	0.267**		0.249**		0.275**	

The number of cases is shown with their percentage within high–stress categories. “High stress” represents participants whose stress response scores are in the top quartile.

Φ: Phi coefficient. Values in bold indicate the absolute value of Φ is more than 0.1.

^*^p < 0.05, ^**^p < 0.001.

A relatively high percentage of tenured and fixed-term teachers indicated a “responsibility for students' learning” as a main stressor (11.4 and 13.4%, respectively); however, the effect sizes of the association with stress responses were negligible (Φ = 0.051 and 0.074, respectively). A relatively high percentage of tenured teachers perceived “school management duties” as a main stressor (15.0%); however, again, the effect sizes of the association with stress responses were negligible (Φ = 0.064). A relatively high percentages of teachers perceived “relationship with co-workers” as a main stressor (8.3–16.8%), and the association with stress responses was practically significant in all job positions (Φ = 0.112–0.155, *p* < 0.001). The percentages of teachers who selected “relationship with supervisors” were not so high (4.8-9.3%); however, the association with high stress responses was practically significant in all job positions, except for principals (Φ = 0.124–0.191, *p* < 0.001). Concerning “relationship with difficult students,” a relatively high percentage of teachers, except for diet and nutrition teachers selected it as a main stressor (16.0–25.6%); however, the effect sizes of the association with stress responses were negligible, except among tenured and fixed-term teachers (Φ = 0.115 and 0.145, respectively). A relatively high percentages of principals, vice-principals, tenured, and fixed-term teachers selected “dealing with challenging parents” as a main stressor (29.1, 22.8, 14.1, and 10.2%, respectively); however, the effect sizes of the association with stress responses was practically significant only in principals and fixed-term teachers (Φ = 0.108 and 0.114, respectively). A high percentage of teachers, except for principals, chose “workload of clerical tasks” as a main stressor (10.6–36.8%). However, the association with stress responses was negligible, except for vice-principals and diet and nutrition teachers (Φ = 0.135 and 0.157, respectively). Moreover, the association between “unfamiliar work environment” and stress responses was negligible in all job positions, except for diet and nutrition teachers (Φ = 0.103). Regarding other main stressor items, the effect sizes of the association with stress responses were negligible in all job positions (Φ < 0.1).

## 4. Discussion

The present study aimed to evaluate work-related stressors affecting teachers' stress responses and clarify main stressors among high-stress teachers. The results revealed that regardless of job positions, quantitative and qualitative workloads, job control, workplace support from supervisors and co-workers are significantly associated with teachers' high stress responses; therefore, Hypothesis 1 was fully supported. The results were consistent with those of previous studies revealing that job demands, job control and social support significantly affected teachers' job burnout and psychological well-being (38, 52). The findings suggest that considering and addressing these factors are important in terms of teachers' work-related stress.

The analysis of interaction effects between job workloads and workplace support showed odd findings. Previous studies support the theory of the JDCS model in which the effects of job demands on teachers' psychological problems, such as depression, anxiety, and burnout, were moderated by job control and workplace support (38, 53). In contrast, the present study did not demonstrate statistically-significant interaction effects between job workloads and job control. The interaction effect between quantitative workload and co-workers' support was statistically significant only for tenured and fixed-term teachers. Furthermore, the results revealed that the odds ratio representing the association between quantitative workload and high stress responses increased with the level of co-workers' support (Table 5). In other words, the deteriorating effects of quantitative workload on teachers' stress responses became more enhanced in the presence of higher co-workers' support, which were totally contradictory findings to those of previous studies. We could not provide reasonable rationale for this odd result at this point. In any case, the theory advocated in the JDCS model was not supported in this study.

The results also revealed that relationships with supervisors and co-workers were important factors influencing teachers' stress responses, regardless of job positions and employment status: their association with teachers' high stress responses was practically significant in all job positions (Φ > 0.1). However, other items, such as dealing with difficult students or parents, workload of clerical tasks and school management duties exhibited practically-significant association with high stress response only for several job positions; therefore, Hypothesis 2 was not fully supported.

Interpersonal problems with other school staff have been reported as one of the most stress-inducing factors among teachers (54–56). Taniguchi et al. revealed that interpersonal stressors were associated with depression among teachers and a negative relationship-oriented coping strategy increased their depression symptoms (57). They also reported that the postponed-solution coping method significantly reduced teachers' depression levels (57). Assertiveness, a social communication style to express oneself openly and honestly while being concerned for others, increases teachers' occupational well-being (58). The effectiveness of these interpersonal coping strategies among teachers have been investigated mainly in the context of teacher-student relationships in previous studies; however, they might be useful for relationship problems experienced with colleagues or supervisors. Thus, considering the importance of interpersonal relationship with co-workers in terms of teachers' occupational stresses, a stress management program which focuses on relationship problems among school staff and communication strategies targeting their relationship with co-workers would be substantially beneficial for teachers' mental health and well-being. A prospective controlled study, which specifically focuses on examining the effectiveness of interpersonal skills training for this purpose, is strongly recommended.

Harassment by supervisors or other employees is also one of the biggest relationship problems in workplaces; it has a serious impact on interpersonal relationships with co-workers. Harassment by colleagues or administrators substantially deteriorates teachers' mental health (59, 60). Developing a comprehensive anti-harassment policy and providing harassment-prevention training is essential to avoid subsequent devastating consequences.

As expected, the results found that long working hours significantly contributed to high stress responses among teachers. Among all teachers' job positions, vice-principals' working hours were substantially the longest. However, their stress response scores were the second lowest next to principals. In contrast, the stress response scores among diet and nutrition teachers and health education teachers were the highest even though their working hours were relatively short. The scores of job control, one of the important stress-reducing factors, were also the highest among diet and nutrition teachers and health education teachers, which also contradicted their high stress response levels. Comparatively, their supervisors' and co-workers' support scores were the lowest among all teachers, meaning that they perceived relatively low levels of workplace support from other school staff. The analysis of these variables suggests that relationship problems with supervisors and co-workers substantially impact teachers' occupational stress.

Relationship problems with difficult students were significantly associated with the stress response levels in tenured and fixed-term teachers, who are mainly responsible for classroom management. This corresponds to previous studies on teachers' stressors (61, 62). To illustrate, a study showed that teachers adopt different coping strategies to students' behavioral problems in relation to their gender, knowledge, and teaching experience (63, 64). Experienced teachers generally know how to cope with difficult students; however, teachers have the chance to encounter a great range of behavioral problems daily which equips them for this (63). Therefore, it would be necessary for teachers to constantly have an opportunity to learn and discuss effective, evidence-based management strategies to handle those students.

Innately, educating students is the key element of educational work and contributes to teachers' active motivation as educators, even toward misbehaving ones. However, because of lack of adequate support from organizations, supervisors and co-workers, an individual teacher may not be able to provide ideal education to students (56). In view of motivation theory, lack of support may downgrade teachers' motivation level, from active motivation to passive one (I would like to educate them, but cannot because of lack of support) or active de-motivation (I do not like to involve them, but must do) (65, 66). This downgrade of teachers' motivation could be associated with a decrease in their levels of *emotioncy*, a newly developed concept in psychology, representing a blend of emotion and frequency of senses involved when individuals experience outer objects or activities (67). Existing literature suggests that the level of emotioncy can affect teachers' emotional labor and burnout (68). Taking measures to encourage teachers to maintain their active motivation toward education, such as providing adequate organizational and supervisors' support concerning misbehaving students' problems, would be required to prevent their burnout.

Regarding workload of clerical tasks, a relatively high percentage of teachers chose this as a main stressor, however, the association with stress response scores was practically significant only among vice-principals and diet and nutrition teachers. Previous studies indicated that paper-work associated with teaching and other peripheral tasks were significantly stressful for teachers (69, 70). Clerical work, such as document preparation and other related duties, constitute approximately 5.6 h per week in Japan, more than double the average for all surveyed countries (20). Increasing the number of support staff who help doing these teachers' peripheral tasks is important to reduce teachers' workload and stress levels.

Public spending on education and school resources can be considerably affected by a country's economic situation. The worldwide economic crisis that began in 2008, being still unresolved, has substantially impacted people's work and living conditions including their mental health in different countries (71). The economic crisis has also affected the function of school units in various ways, creating inequality in children's education, reducing training opportunities for teachers, and causing a shortage of school staff and resources (72). The Japanese economy has also been significantly impacted by the global economic crisis and not yet recovered in a sustained fashion (73). The coronavirus disease 2019 (COVID-19) has also inflicted a severe shock to global economy (74). The Japanese economy has been experiencing serious difficulties because of the pandemic (75). This long-standing severe economic situation in Japan will be affecting the fulfillment of support system for public school teachers in this country in a negative way.

As previously described, working hours of school teachers in Japan are reported to be the highest among the OECD member countries (20). One reason for their severe working conditions is the relatively large class size in schools in Japan. The student-teacher ratio is much higher in Japan compared with other OECD member countries (76). The lack of sufficient number of teachers compared to the number of students will affect the quality of education, teachers' job satisfaction, and occupational stress, possibly influencing their relationship with supervisors and co-workers indirectly. Considering the high student-teacher ratio in Japan, increasing the number of school teachers will be required to protect teachers' mental health.

Public spending on primary to tertiary education in 2019 was 7.8% of the total government expenditure in Japan, which was lower than the OECD average (10.6%) (76). It can be noted that Japanese economic situation is still severe, however, considering the relatively small public expenditure on education in Japan, the government should spend more money on children's education, school resources, and increasing teachers and support staff.

Although this study provided several important insights, it also had some limitations. The study adopted a cross-sectional research design; thus, the causality of relationships between stress responses, long working hours, and stressors was unclear. This study investigated occupational stressors among primary school teachers. In different school settings, such as high schools or special education schools, the results may be different. To illustrate, previous studies reported that job satisfaction, role conflicts and ambiguities, and other buffering factors were related to teachers' psychological stressors (77–79). The study was conducted in 2021 when COVID-19 was still spreading in Japan; therefore, it might have affected the present results. Further well-designed, prospective studies incorporating these variables are required to address these possible biases.

The present study aimed to identify the main stressors among highly-stressed primary school teachers by using large-scale national-level survey, considering the difference between different job positions. Quite remarkably, the results demonstrated that relationships with supervisors and co-workers were substantially associated with stress response levels among teachers, regardless of job positions. Interpersonal problems with school staff have been reported as one of the stress-inducing factors among teachers (54–56), however, the present study further highlighted the role of teachers' relationship issues with co-workers. In this regard, despite the above-mentioned limitations, we believe the study will provide useful insights and proposals into this field of research.

## 5. Conclusion

The present study investigated main stressors among high-stress primary school teachers in Japan. Regardless of job position, relationships with supervisors and colleagues were significantly associated with stress response levels among teachers. Dealing with difficult students and parents, and workloads of clerical tasks were also associated with teachers' stress responses depending on job position. These findings suggest that providing interpersonal skills training targeting co-workers' relationships and harassment prevention measures are crucial to maintain teachers' mental health. Additionally, the results suggest that increasing of school teachers and support staff and providing sufficient organizational and supervisors' support would be required to prevent teachers' burnout.

## Data availability statement

The datasets presented in this article are not readily available because we cannot publicly provide individual data due to a data provider's regulations. Qualifying researchers may apply to access a minimal dataset upon reasonable request by contacting the corresponding author. Requests to access the datasets should be directed to tubonok@tokaihp.jp.

## Ethics statement

The studies involving human participants were reviewed and approved by Tokai Central Hospital. Written informed consent for participation was not required for this study in accordance with the National Legislation and the Institutional Requirements.

## Author contributions

KT contributed to the design, implementation of the research, analysis of the results, and writing of the manuscript. MO critically reviewed the manuscript and supervised the whole research process. Both authors critically revised the report, commented on drafts of the manuscript, and approved the final report.
